# Premature Ejaculation after Lithium Treatment in a Patient with Bipolar Disorder

**DOI:** 10.1155/2023/6156023

**Published:** 2023-01-09

**Authors:** Pedro Amadeu Almeida, Filipa Caldas, Inês Homem de Melo, Ana Maria Moreira, Gustavo França Santos

**Affiliations:** Psychiatry Department, Hospital de Magalhães Lemos, Porto, Portugal

## Abstract

Lithium has proven its efficacy in treating bipolar disorder. Severe side effects caused by lithium, including renal and endocrine outcomes, have already been amply documented. The impact of lithium on sexual function, however, is less well known. A 33-year-old man, with no past medical history, diagnosed with bipolar disorder, developed premature ejaculation after short-term use of lithium. The dose of lithium was reduced, leading to a rapid clinical resolution. Retrospectively, lithium-induced premature ejaculation was deemed the most likely diagnosis. Premature ejaculation is a rare side effect of lithium. Changing the time of medication administration and lowering dose could be considered as alternatives. Given lithium's pharmacological profile, it is likely that the pathophysiologic mechanism behind premature ejaculation is altered levels or altered serotonin receptor sensitivity in the ejaculatory modulating centers of the central nervous system. Given the reluctance to spontaneously report sexual adverse effects, clinicians should be aware of this possible side effect.

## 1. Introduction

Lithium remains the first-line treatment for bipolar disorder, having the strongest evidence for long-term relapse prevention. Controlled trials provide support for this effectiveness, showing fewer recurrences of depression and effects on suicide and deliberate self-harm prevention [1]. Nevertheless, lithium is not devoid of significant side effects. The risk of progressive renal impairment is well established, while endocrine, metabolic, and gastrointestinal outcomes have also been extensively studied [2]. There are, however, a limited number of studies assessing sexual side effects in patients on lithium [3].

Premature ejaculation (PE) is the most prevalent male sexual dysfunction [4]. According to the Diagnostic and Statistical Manual of Mental Disorders 5th Edition (DSM-5), PE is defined by the inability to control or delay ejaculation, which always or nearly always occurs before or within about one minute of vaginal penetration [5]. It is associated with negative outcomes such as dissatisfaction or distress for the patient with potential avoidance of sexual intimacy. Multiple studies have shown that several drugs could increase the risk of PE [6].

We report a case of premature ejaculation induced by lithium that, to our knowledge, is the first described in the medical literature. To this end, a review of the pharmacological action of lithium will be carried out in order to investigate the biological mechanisms that may explain its association with PE.

## 2. Case Presentation

A 33-year-old man with bipolar disorder stabilized with quetiapine 150 mg/day was followed in our psychiatry clinic. Considering the high risk of recurrent depressive or manic episodes, we decided to start a proper mood stabilizer.

On 14 February 2020, lithium 400 mg was introduced, and on 7 March 2020, it was steadily increased up to 1000 mg/day. On 26 May 2020, on the next medical appointment, the patient started to report PE. He stated that over the past month, the intercourse time decreased, and the orgasm was achieved within one minute of penetration. This experience was profoundly distressing and present on all instances of sexual activity. Neither prominent erections nor sexual arousal was present. He denied erectile dysfunction, fever, and prostatitis-like symptoms. He had tried topical anesthetics bought in a pharmacy over the counter without any improvement. In the past, he never had any complaint about sexual function, and even when depressed, he had only mildly diminished libido but with normal erection and ejaculation. When manic, he reported increased libido but with no adverse behavioral outcomes.

At this time, the patient was on quetiapine 150 mg per day and lithium 1000 mg per day, only. He had not developed any other side effect with lithium treatment, such as signs of acute intoxication. The patient did not report any perceived stressor preceding the current episode. He had no medical comorbidities such as diabetes, hypertension, or dyslipidemia. There was no history of drug abuse.

The patient's vitals were stable on presentation. On the mental state examination, the patient was euthymic, with appropriate and congruent affect, but concerned about the present symptom. His thought process was linear and coherent. The remaining physical and neurological examinations were normal.

All routine blood investigations including complete blood count, renal, liver, electrolytes, and thyroid function tests were unremarkable. His serum lithium levels increased from 0.4 mmol/L (on March 21^st^ 2020, with a lithium dosage of 400 mg/d) to 0.81 mmol/L (on June 5^th^ 2020, with a lithium dosage of 1000 mg/d). On the 5^th^ of June 2020, serum levels of prolactin were 29.4 ng/mL (lab reference values: 4.04-15.2).

After careful evaluation, dapoxetine 60 mg was prescribed and, at the same time, psychoeducation and behavioral treatment with pause-squeeze techniques were delivered. In the following consultation, the patient told us he did not take dapoxetine, concerned about potential side effects. Lithium was then gradually reduced from 1000 mg/d to 400 mg/d, and within one week, the patient reported normalization of his sexual activity. In the follow-up visits, the patient remained asymptomatic. Despite the reduction of lithium, the patient remained mentally stable.

Following clinical resolution, on the 21^st^ of October 2020, lithium was 0.32 mmol/L (with a lithium dosage of 400 mg/d) and serum prolactin levels decreased to normal levels. Given his age, the absence of comorbidities and the temporal association between PE and the commencement of lithium, and its successful resolution following reduction, lithium-induced PE was deemed the most likely diagnosis.

## 3. Discussion

Sexual dysfunctions in men are a heterogeneous group of disorders which encompass anomalies with desire, erectile function, and ejaculation. A wide range of psychotropic drugs can cause sexual dysfunctions, compromising treatment compliance [7].

Lithium's impact on sexual function is appreciable at different clinical levels. Although more studies are needed, it seems to have an adverse effect on sexual desire and erectile function [3, 8, 9] which paradoxically could be useful in a severe mental disorder where hypersexuality is occasionally found [10]. Cotherapy with benzodiazepines may increase the risk of sexual dysfunction [11], which correlates with a lower level of functioning and poor medication compliance [12]. The nitric oxide pathway seems to play a role in erectile dysfunction during lithium treatment [3]; therefore, phosphodiesterase type 5 inhibitors (such as sildenafil) could be used [13]. Less is known about lithium's impact on ejaculation.

Ejaculation is centrally mediated by a variety of brain structures, including anterior thalamic nuclei and the medial preoptic area of the hypothalamus, with many neurotransmitters involved including serotonin (5-HT), dopamine, oxytocin, gamma-aminobutyric acid (GABA), adrenaline, and nitric oxide. Overall, ejaculation is facilitated by dopamine and inhibited by 5-HT [14]. 5-HT is one of the most studied neurotransmitters in the neurophysiology of ejaculation, and three 5-HT receptor subtypes (5-HT_1A_, 5-HT_1B_, and 5-HT_2C_) have been postulated to mediate 5-HT's activity on ejaculation [15]. The complex nature of the ejaculatory reflex makes the definition of the precise role of each neurotransmitter difficult. Nevertheless, it is likely that premature ejaculation is caused by hyposensitivity of 5-HT_2C_ receptors and hypersensitive 5-HT_1A_ receptors [14]. At the neuronal level, it is held that lithium has a net-enhancing effect on 5-HT function with several studies showing lithium's blocking effect on the 5HT_2C_ receptors [16, 17].

Lithium's effect on dopamine signaling pathways is poorly understood, but overall, it is believed that dopamine neurotransmission is inhibited through the modulation of G-protein-coupled dopamine receptors [18]. Hyperprolactinemia is associated with reduced sexual desire, erectile, or ejaculatory dysfunction, including either premature or more commonly delayed ejaculation [19]. The lack of decreased sexual desire or erectile dysfunction suggests that the effect on 5-HT receptor sensitivity in the ejaculatory modulating centers of the central nervous system including the activation of the 5-HT_1A_ receptor and the blockade 5-HT_2C_-induced by lithium, rather than altered dopaminergic transmission, is the most likely explanation for this side effect.

PE increases the already high burden of bipolar disorder with added psychological suffering and interpersonal stress. Men with PE report increased anxiety and interpersonal difficulties [20]. Moreover, this side effect raises concern over treatment compliance with a potential impact on the natural course of the disorder.

**Figure 1:**
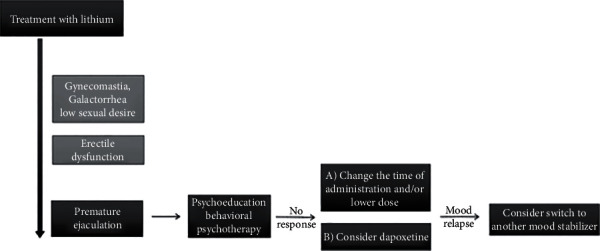
Management of premature ejaculation induced by lithium.

Lithium-induced PE requires a careful assessment of the benefits and risks of therapy. Treatment could include psychoeducation, behavioral psychotherapy, and pharmacotherapy [21]. Dapoxetine is the only drug approved for on-demand treatment of PE in Europe [22]. However, it has substantial side effects, including reduced libido. The risk of manic relapse, though unlikely, is plausible. Furthermore, serotonergic syndrome albeit rare is potentially fatal and may occur with concomitant use of lithium [23]. When changing to a different mood stabilizer is not an option, several strategies could be considered, including lowering dose and adjusting the time of medication administration (to after sexual intercourse) (see Figure 1).

Psychoeducation and behavioral psychotherapy (including start-stop method and squeeze techniques) are the mainstays of treatment. In case of no response, several strategies could be considered, including the following:

- Lowering dose and adjusting the time of medication administration to after sexual intercourse, and
- Adding dapoxetine, which is the only drug approved for on-demand treatment of PE in Europe. Both approaches could increase the risk of bipolar disorder relapse. Albeit rare, dapoxetine also enhances the risk of a serotonergic syndrome, which is potentially fatal

After lithium steadily increased from 400 mg/day to 1000 mg/day, our patient developed PE. This report calls attention to a dose-dependent effect. The risk of PE induced by lithium is also likely to interact with the patient's age; psychological variables, such as temperament and personality; and medical comorbidities. Whether this effect is transitory, as some of the acute sexual side effects induced by psychotropics, or long-lasting remains unclear. The risk of PE during long-term lithium treatment must be weighted with thyroid function changes induced by lithium, as thyroid disorders could be related to PE [24].

A limitation of this case report is the concomitant administration of quetiapine, a dopaminergic antagonist, which is known to have sexual side effects. A pharmacokinetic interaction between lithium and quetiapine is implausible as their elimination routes are very different. Still, we cannot exclude a pharmacodynamic interaction between lithium and quetiapine. Quetiapine, as a dopamine receptor antagonist, could enhance the ejaculatory threshold, lowering the risk of premature ejaculation. Nevertheless, quetiapine was already prescribed before lithium commencement and it was not changed.

Another limitation is related to the clinical diagnosis of premature ejaculation, which relies only on phenomenological presentation, lacking objective data assessments. It also provides space for discussion of confounding cultural factors, regarding expectations or prohibitions about the experience of sexual pleasure.

In conclusion, some factors point to a specific relationship between lithium and the development of PE in this patient: temporal criterion (administration of lithium shortly before the onset of PE), biological plausibility (effects of lithium on serotonin receptors), and dose-related criterion (the decrease in the lithium dose soon followed by the improvement in PE). Although uncommon, we consider it important that psychiatrists are informed about this adverse effect given that sexual dysfunctions, including PE, are one of the main reasons for discontinuing psychotropic drugs prescribed for psychiatric conditions [25]. As this discontinuation is often unreported by patients, a systematic questioning of sexual problems is recommended when prescribing lithium.

## Data Availability

Data sharing is not applicable to this article as no new data were created or analyzed.

## Abbreviations

DSM-5: Diagnostic and Statistical Manual of Mental Disorders 5th Edition (DSM-5)
PE: Premature ejaculation.

## Additional Points

*Highlights*. (1) Acquired PE may be a dose-dependent side effect of lithium therapy. (2) Given lithium's pharmacological profile, it is likely that the pathophysiologic mechanism behind premature ejaculation is altered levels or altered 5-HT receptor sensitivity in the ejaculatory modulating centers of the central nervous system. (3) During follow-up of patients on lithium therapy, it is essential to ascertain the presence of any sexual adverse effects. PE is likely to be underreported and undertreated because of the patient's perceived shame and embarrassment.
